# Prebiotic Activity of *Acorus gramineus* Rhizome Extract and Its Effects on Obesity and Gut Inflammation

**DOI:** 10.1002/fsn3.70020

**Published:** 2025-01-30

**Authors:** Hye‐Bin Lee, Mi‐Jin Oh, Hee‐Kyoung Son, Miri Park, Sang Yoon Choi, Jinyoung Hur, Ho‐Young Park

**Affiliations:** ^1^ Food Functionality Research Division Korea Food Research Institute Wanju‐gun Jeollabuk‐do Republic of Korea; ^2^ Department of Food Biotechnology Korea National University of Science and Technology Daejeon Republic of Korea

**Keywords:** anti‐obesity, gut permeability, intestinal inflammation, prebiotic activity, sweet flag rhizome

## Abstract

*Acorus gramineus* (sweet flag), a medicinal plant, especially its rhizome, shows strong antioxidant and anti‐inflammatory effects; however, its efficacy in treating intestinal inflammation and obesity is still unexplored. In this study, we investigated the prebiotic activity of sweet flag rhizome extract (SRE) and its preventive effects against high‐fat diet (HFD)‐induced obesity and colonic inflammation. The prebiotic activity was assessed based on the prebiotic activity scores of four probiotic strains. SRE was administered to mice fed an HFD for 8 weeks, and obesity‐ and inflammation‐related biomarkers were analyzed. The results showed that SRE was utilized as effectively as inulin by the probiotic strains, suggesting that SRE has the potential to influence the gut. Additionally, the administration of 100 mg/kg SRE mitigated colonic inflammation by restraining gut permeability, endotoxemia, and colonic shortening, and improved gut barrier function by restoring zonula occludens‐1 protein expression. SRE ameliorated obesity‐related symptoms by suppressing weight gain, glucose intolerance, serum lipid biomarkers, and liver damage. Altogether, this study highlights the protective effects of SRE against obesity and intestinal inflammation and provides data for the further application of SRE as a prebiotic.

## Graphical abstract

This study investigated the prebiotic activity of sweet flag ( *Acorus gramineus* ) rhizome extract (SRE) and its preventive effects against high‐fat diet (HFD)‐induced obesity and colonic inflammation. The results showed that SRE was utilized as effectively as inulin by the probiotic strains suggesting that SRE has the potential to influence the gut. In addition, the administration of 100 mg/kg SRE improved HFD‐induced colonic inflammatory symptoms and also attenuated obesity‐related symptoms by inhibiting weight gain, glucose intolerance, and serum lipid biomarkers.

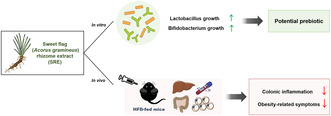

## 1 Introduction

Modern dietary patterns, including a high‐fat diet (HFD) and a Western‐style diet, increase inflammation throughout the body, leading to a variety of diseases (Clemente‐Suárez et al. 2023). Obesity caused by these dietary patterns is a major problem worldwide and is closely related to diseases, such as hypertension, diabetes, and cardiovascular disease (Ji et al. 2023). Managing obesity safely and effectively to reduce mortality risk and improve the quality of life has become a topic of concern for researchers (Yang et al. 2024).

In humans, HFD also influences the pathogenesis of intestinal diseases, such as intestinal hyperpermeability and inflammatory bowel disease (Rohr et al. 2020). HFD‐induced gut dysbiosis increases intestinal barrier permeability, and the resulting elevated lipopolysaccharide levels cause metabolic endotoxemia and intestinal inflammation (Violi and Nocella 2023). Dietary modifications can modulate the gut microbiota, thereby improving gut barrier function and suppressing endotoxin translocation into circulation (Ranneh et al. 2023). Therefore, the consumption of prebiotics, probiotics, and plant‐based diets is considered a potential therapeutic strategy (Li et al. 2020; Pérez‐Burillo et al. 2020).

The genus *Acorus* is an aromatic plant known for its medicinal properties and has a long history of use in India and China (Rajput, Tonge, and Karuppayil 2014). The species of *Acorus* are divided into two natural groups, of which the *A. gramineus* group grows mainly in East and Southeast Asia (Sokoloff et al. 2024). Various chemical constituents have been reported from the rhizome and leaves of *A. gramineus* , including β‐asarone, α‐asarone, and essential oil (Zhao et al. 2023). Among the plants of the genus *Acorus*, the rhizome extracts of *A. calamus* have been reported to exhibit anti‐inflammatory, antioxidant, and anticancer effects, but studies on the efficacy of *A. gramineus* have not been sufficient (Rajput, Tonge, and Karuppayil 2014; Umamaheshwari and Rekha 2018; Zhao et al. 2023). In addition, the anti‐obesity and gut‐health‐promoting effects of sweet flag rhizome extract (SRE) have not been sufficiently explored. In this study, we investigated the in vitro prebiotic activity of SRE, and its anti‐obesity and gut inflammation‐alleviating effects in HFD‐fed mice.

## 2 Materials and Methods

### 2.1 Extract of Sweet Flag Rhizome

Dried sweet flag ( *A. gramineus* ) rhizomes were ground into powder and extracted with 20 volumes of distilled water at 80°C for 3 h. The extracted sample was then filtered, concentrated under reduced pressure, and freeze‐dried to obtain SRE, which was stored at −20°C for subsequent use.

### 2.2 Determination of Chemical Composition

Total sugar analysis was performed using the phenol‐sulfuric acid assay as previously described (Lee et al. 2020), using glucose as a standard and measuring the absorbance at 470 nm. Protein content was analyzed using a Bio‐Rad kit (DC protein assay kit; Hercules, CA, USA), according to the manufacturer's protocol. The total phenolic content was analyzed using Folin–Ciocalteu's colorimetric method (Zhao et al. 2008), using gallic acid as a standard and measuring the absorbance at 750 nm. Total flavonoid analysis was performed using quercetin as a standard with slight modifications (Cosmulescu, Trandafir, and Nour 2017). Briefly, 0.1 mL diluted standard solutions or extracts was mixed with 0.1 mL of 10% aluminum nitrate, 0.1 mL of 1 M potassium acetate, and 4.7 mL of 80% methanol. After keeping it in a dark room for 45 min, the absorbance was measured at 415 nm.

### 2.3 Prebiotic Activity of SRE

The probiotics *Lactobacillus bulgaricus* , *Lactobacillus gasseri* , *Bifidobacterium longum* , and *Bifidobacterium bifidum* were obtained from Mediogen (Chungju, Republic of Korea), and *Escherichia coli* was purchased from the Korean Collection for Type Cultures (KCTC2441; Jeongup, Republic of Korea). We performed the prebiotic activity assay as described previously (Lee et al. 2021). First, probiotic strains were cultured in de Man, Rogosa, and Sharpe (MRS) agar and MRS broth, and *E. coli* was cultured in tryptic soy agar and tryptic soy broth. Strains grown in broth were centrifuged and suspended in M9 broth supplemented with 0.1% glucose, 0.05% MgSO_4_, and 0.0015% CaCl_2_ to obtain an absorbance of 2.50 at 600 nm. The bacterial culture medium adjusted by absorbance was inoculated into 0.5% glucose, 0.5% inulin, 0.1% SRE, and 0.5% SRE dissolved in M9 broth, and cultured at 37°C for 24 h. The score was calculated using the equation below (Fara et al. 2020):

$$\begin{array}{c} \text{Prebiotic activity}\;\text{score}=\frac{\text{probiotic}\;\text{biomass}\;\mathrm{on}\;\text{the}\;\text{prebiotic}\;\mathrm{at}\;24\;h-\text{probiotic}\;\text{biomass}\;\mathrm{on}\;\text{the}\;\text{prebiotic}\;\mathrm{at}\;0\;h}{\text{probiotic}\;\text{biomass}\;\mathrm{on}\;\text{glucose}\;\mathrm{at}\;24\;h-\text{probiotic}\;\text{biomass}\;\mathrm{on}\;\text{glucose}\;\mathrm{at}\;0\;h}-\\ \frac{\text{enteric}\;\text{biomass}\;\mathrm{on}\;\text{the}\;\text{prebiotic}\;\mathrm{at}\;24\;h-\text{enteric}\;\text{biomass}\;\mathrm{on}\;\text{the}\;\text{prebiotic}\;\mathrm{at}\;0\;h}{\text{enteric}\;\text{biomass}\;\mathrm{on}\;\text{glucose}\;\mathrm{at}\;24\;h-\text{enteric}\;\text{biomass}\;\mathrm{on}\;\text{glucose}\;\mathrm{at}\;0\;h} \end{array}.$$

### 2.4 Experimental Design for Animal Study

Forty‐five male C57BL/6N mice aged 6 weeks were purchased from Orient Bio (Seongnam, Republic of Korea). After 1 week acclimation, mice were randomly divided into five groups: (i) ND group: normal chow diet (2918C; Envigo RMS, Indianapolis, IN, USA) and sterilized water administration; (ii) HFD group: HFD (TD.06414; Harlan, Madison, WI, USA) and sterilized water administration; (iii) positive control (PC) group: HFD and fructooligosaccharide 50 mg/kg/day administration; (iv) SRE50 group: HFD and SRE 50 mg/kg/day administration; and (v) SRE100 group: HFD and SRE 100 mg/kg/day administration. All the interventions were maintained for 8 weeks. Body weight was recorded weekly, and body fat mass, oral glucose tolerance test (OGTT), and gut permeability were performed at 8 weeks of feeding. Fat mass was measured using a dual‐energy *x*‐ray asorptiometry (InAlyzer, Medikors, Seongnam, Republic of Korea). Fat tissue is shown in red and lean is in green. After 8 weeks, all the mice were sacrificed under isoflurane anesthesia, and serum, white adipose tissue (WAT), liver, and colon samples were collected. WAT and liver tissues were weighed, and colon tissues were measured for length and pH. Colon pH was analyzed by flushing the internal contents with sterile water and measuring the pH using a pH meter (Orion Star A211; Thermo Fisher Scientific, Waltham, MA, USA). All experimental protocols were approved by the Korea Food Research Institutional Animal Care and Use Committee (KFRI‐M‐22038).

### 2.5 Serum Biomarker Analysis

Serum triglycerides, total cholesterol, high‐density lipoprotein (HDL) cholesterol, low‐density lipoprotein (LDL) cholesterol, alanine aminotransferase (ALT), and aspartate transaminase (AST) were measured using an automatic analyzer (Hitachi 7180, Tokyo, Japan). Serum endotoxin was measured using a commercial kit (Pierce Chromogenic Endotoxin Quant Kit, A39552; Thermo Fisher).

For OGTT experiments, mice were fasted for 15 h, and their fasting glucose levels were measured using a glucometer (Accu‐Chek Performa; Roche, Basel, Switzerland). Glucose (2 g/kg body weight) was then administered, and glucose levels were measured at 30, 60, 90, and 120 min.

For gut permeability experiments, mice were fasted for 5 h, and FD4 (500 mg/kg body weight; Sigma‐Aldrich, St. Louis, MO, USA) was administered. Blood was sampled at 2 and 5 h in K3 EDTA tubes (SARSTEDT, Nümbrecht, Germany).

Blood was immediately centrifuged, and FD4 levels in plasma were analyzed using a microplate reader (Molecular Devices, Sunnyvale, CA, USA) at excitation wavelength 485 nm and emission wavelength 535 nm.

### 2.6 Western Blot Analysis

Colon was homogenized with tissue protein extraction reagent (78,510; Thermo Fisher Scientific). Lysates containing 20 μg protein were loaded and separated on sodium dodecyl sulfate‐polyacrylamide gels. After electrophoresis, proteins were transferred to polyvinylidene fluoride membranes. Then, the membranes were blocked with 5% skim milk, incubated with primary antibodies, and then with secondary antibodies at 4°C. Antibodies targeting zonula occludens‐1 (ZO‐1) and occludin were purchased from Abcam (Cambridge, UK); claudin1 was purchased from Thermo Fisher Scientific. Membranes were visualized with an ChemiDoc XRS+ imaging system (Bio‐Rad).

### 2.7 Statistical Analysis

Data are expressed using GraphPad Prism version 10.2.1 (GraphPad Software, San Diego, CA, USA) and IBM SPSS Statistics version 20 (IBM SPSS, Armonk, NY, USA). For group comparisons, one‐way analysis of variance was performed and significance determined using Duncan's multiple range test. A *p* values < 0.05 was considered statistically significant, and results are shown as mean ± standard deviation (in vitro) and mean ± standard error of the mean (in vivo).

## 3 Results

### 3.1 Chemical Composition of SRE

SRE was obtained through hot water extraction, and its chemical composition was determined (Table 1). The extraction yield was 21.9% (w/w). The total sugar and protein contents were 713.7 and 154.3 mg/g, respectively, which accounted for most of the SRE. In addition, the polyphenol and flavonoid contents were 27.4 and 10.0 mg/g, respectively.

**TABLE 1:** Yield and chemical composition of sweet flag rhizome extract (SRE).

	Value
Extraction yield of SRE (%)	21.9
Chemical composition of SRE (mg/g)	
Total sugar	713.7 ± 6.6
Protein	154.3 ± 33.8
Polyphenol	27.4 ± 0.5
Flavonoid	10.0 ± 1.8

*Note:* Data are presented as the mean ± standard deviation (*n* = 3).

### 3.2 Prebiotic Activity of SRE

**FIGURE 1:**
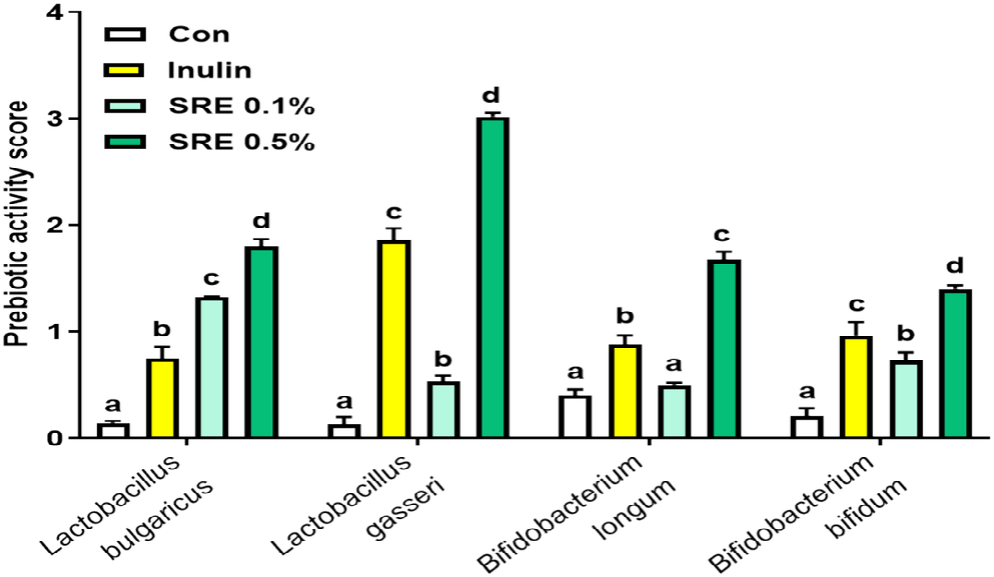
Prebiotic activity of sweet flag rhizome extract (SRE) in probiotic bacteria after incubation for 24 h. The data are expressed as mean ± standard deviation (*n* = 3), and values with different letters (a–d) indicate significant differences at *p* < 0.05 using Duncan's multiple range tests.

To investigate the effect of SRE on prebiotic activity, we evaluated the prebiotic activity scores using four probiotic strains (Figure 1). Overall, for the four strains, the SRE group had significantly higher prebiotic activity scores than the control group (*p* < 0.05). The prebiotic activity score of SRE increased in a concentration‐dependent manner; in particular, the SRE 0.5% group showed significantly higher scores than the positive control inulin group for all four probiotic strains (*p* < 0.05).

### 3.3 Effects of SRE on Obesity‐Related Metabolic Dysfunctions in HFD‐Induced Mice

**FIGURE 2:**
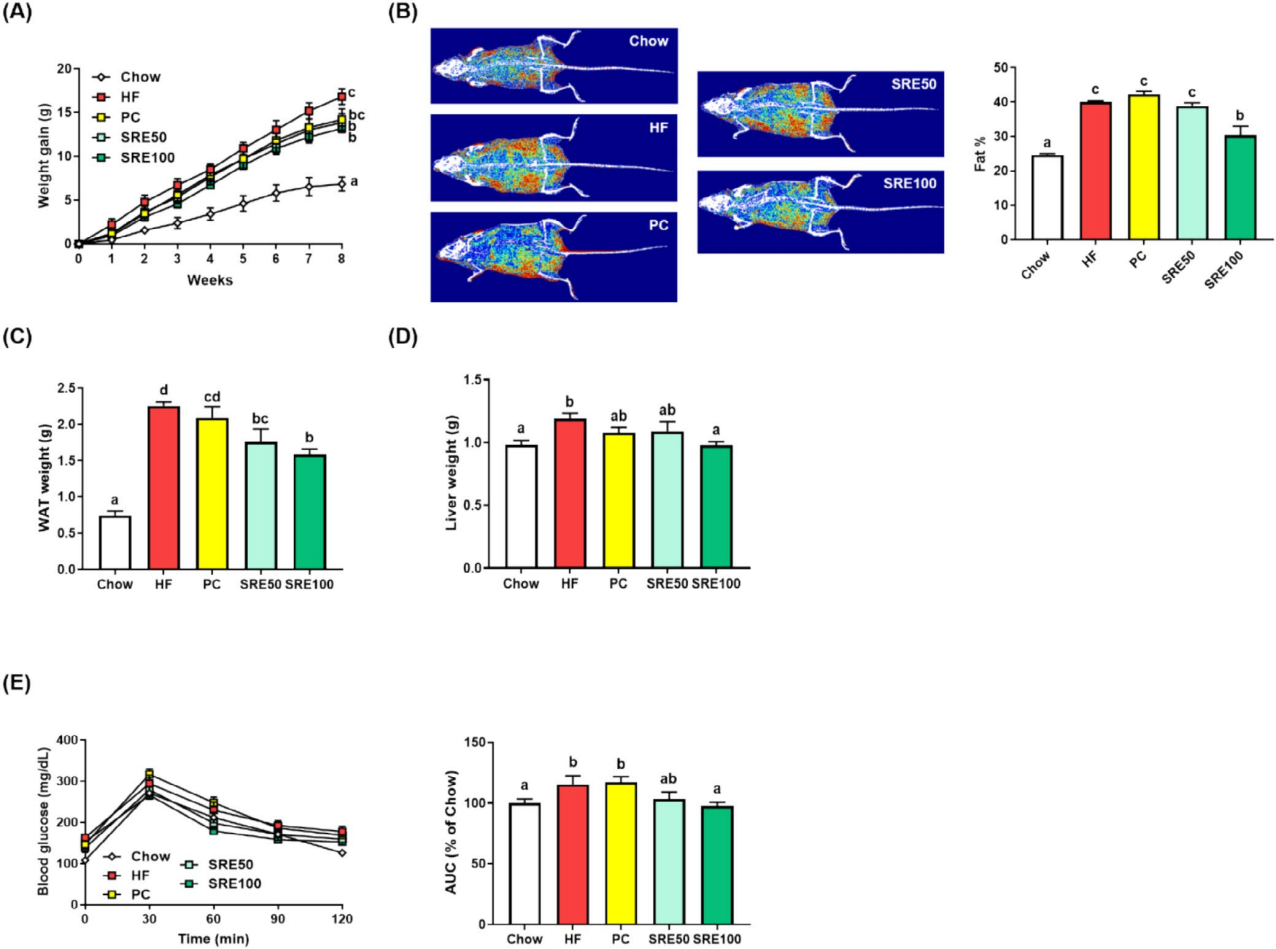
Sweet flag rhizome extract (SRE) suppressed high‐fat diet‐induced gain in body and tissue weights, fat content, and hyperglycemia in obese mice. Changes in (A) body weight gain; (B) body composition images (fat tissue is shown in red and lean is in green) obtained using dual‐energy *x*‐ray absorptiometry and fat percentage; (C) white adipose tissue (WAT) weight; (D) liver weight; and (E) blood glucose levels during glucose tolerance tests and area under the curve (AUC). The data are expressed as mean ± standard error of the mean (*n* = 9), and values with different letters (a–d) indicate significant differences at *p* < 0.05 using Duncan's multiple range tests.

Eight weeks after starting feeding, we observed that the SRE‐administered groups had significantly suppressed body weight gain compared to the HFD group (*p* < 0.05; Figure 2A). The administration of SRE 100 mg/kg reduced body fat mass, WAT weight, and liver weight in HFD‐fed mice (*p* < 0.05, Figure 2B–D). Additionally, OGTT analysis revealed that SRE administration significantly decreased HFD‐induced glucose intolerance (*p* < 0.05; Figure 2E).

**FIGURE 3:**
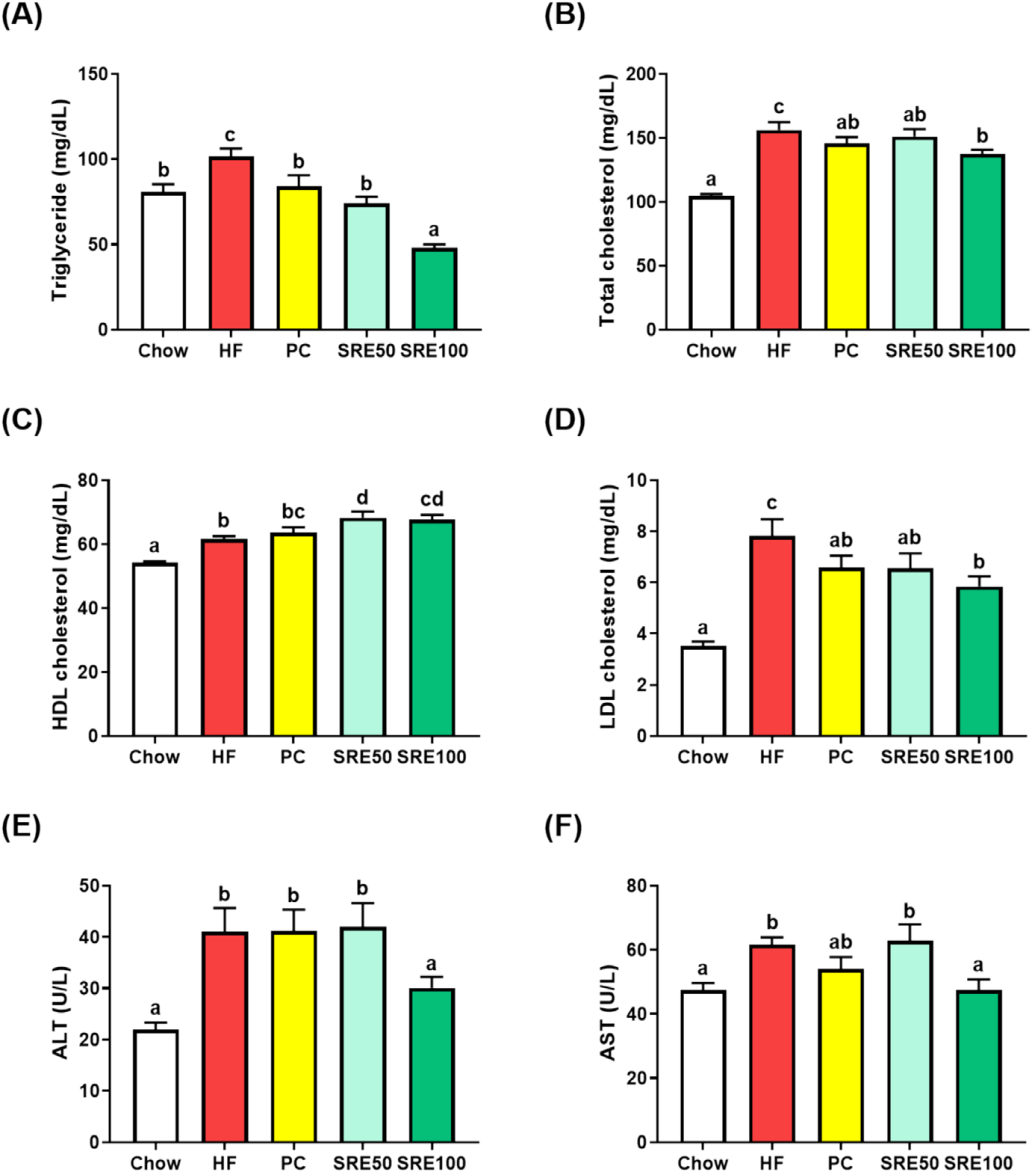
Sweet flag rhizome extract (SRE) suppressed high‐fat diet‐induced serum biomarker levels of lipid parameters and liver function in obese mice. Changes in (A) triglyceride; (B) total cholesterol; (C) high‐density lipoprotein (HDL) cholesterol; (D) low‐density lipoprotein (LDL) cholesterol; (E) alanine aminotransferase (ALT); and (F) aspartate aminotransferase (AST) levels. The data are expressed as the mean ± standard error of the mean (*n* = 9), and values with different letters (a–d) indicate significant differences at *p* < 0.05 using Duncan's multiple range tests.

In addition, we analyzed serum biomarkers including triglycerides, total cholesterol, HDL cholesterol, LDL cholesterol, ALT, and AST (Figure 3). The levels of serum lipid biomarkers triglyceride, total cholesterol, and LDL cholesterol were significantly reduced following SRE administration during the HFD (*p* < 0.05, Figure 3A,B,D); in contrast, HDL cholesterol levels were significantly increased (*p* < 0.05, Figure 3C). The levels of fatty liver disease biomarkers ALT and AST were also lower in the SRE100 group than in the HFD group (*p* < 0.05, Figure 3E,F).

### 3.4 Effects of SRE on Metabolic Endotoxemia and Gut Permeability in HFD‐Induced Mice

**FIGURE 4:**
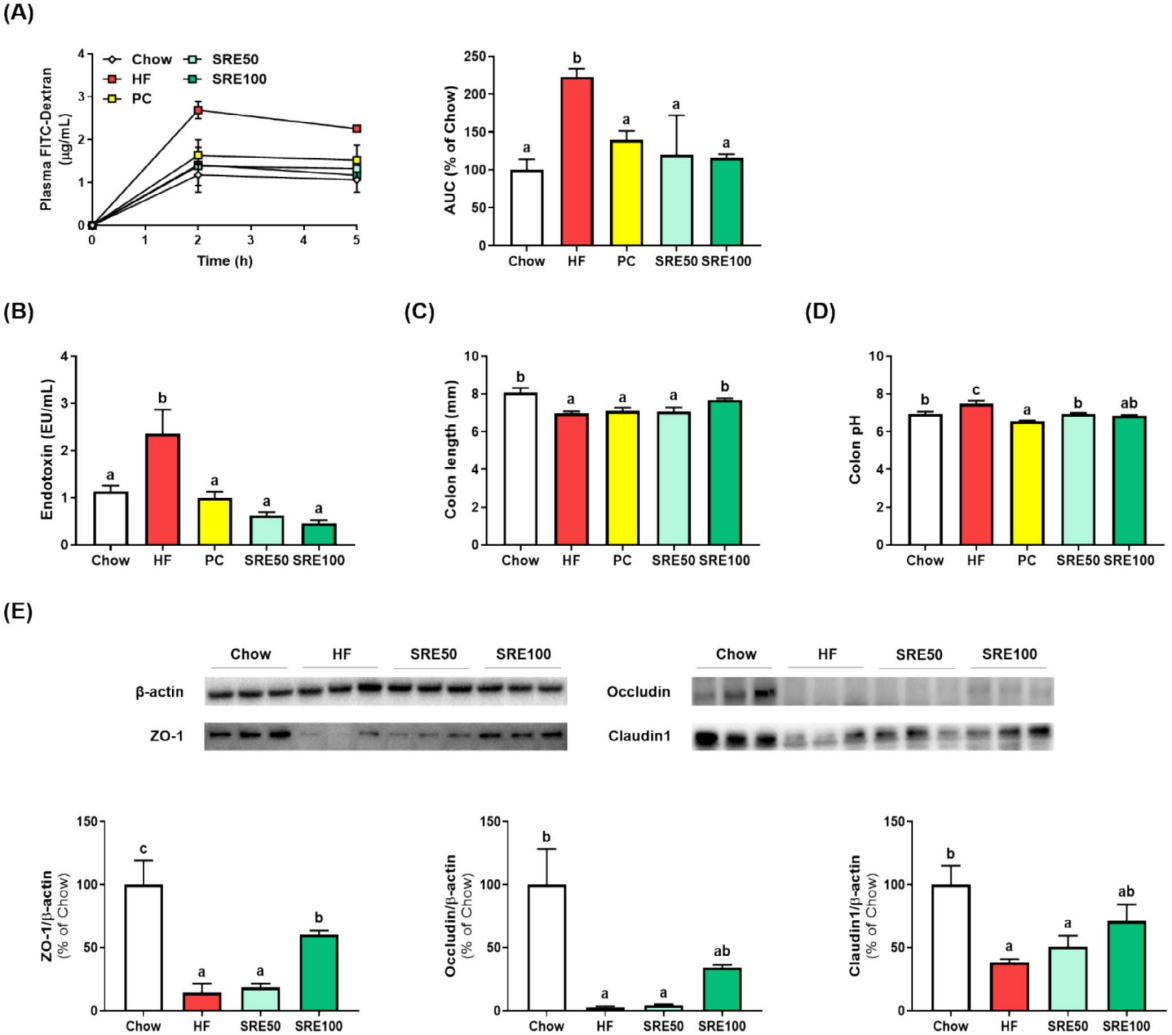
Sweet flag rhizome extract (SRE) reversed high‐fat diet‐induced changes in gut environment and gut barrier function in obese mice. (A) Plasma fluorescein isothiocyanate (FITC)‐dextran levels during permeability tests and area under the curve (AUC); (B) serum endotoxin; (C) colon length; (D) colon pH; and (E) expression of tight junction proteins in colon tissue. The data are expressed as the mean ± standard error of the mean (*n* = 9), and values with different letters (a–c) indicate significant differences at *p* < 0.05 using Duncan's multiple range tests.

Eight weeks after starting feeding, we analyzed gut permeability using FD4 gavage and observed that the SRE‐administered groups had a significantly reduced area under the curve compared with the HFD group (*p* < 0.05, Figure 4A). In addition, SRE administration suppressed serum endotoxin levels in HFD (*p* < 0.05; Figure 4B). HFD induced shortening of colon length and increased pH, whereas SRE administration increased colon length and decreased pH in HFD‐fed mice (*p* < 0.05, Figure 4C,D). Changes in gut barrier integrity were investigated through the expression of tight junction proteins (Figure 4E), which tended to increase with SRE administration in HFD‐fed mice. However, no significant difference was observed, except in ZO‐1 expression.

## 4 Discussion

Obesity, which is caused by the excessive accumulation of body fat, is a complex chronic disease that can lead to negative health effects, such as cardiovascular disease, diabetes, and cancer (Jung et al. 2024). Many studies have reported that obesity and intestinal inflammation are positively correlated with an impaired intestinal barrier and increased levels of pro‐inflammatory factors (Bona et al. 2022; Rohm et al. 2021). Human trials have shown changes in the gut microbial composition in obese patients (Van Hul and Cani 2023), which have been reported to cause metabolic endotoxemia and intestinal inflammatory responses (Cani et al. 2009). To overcome these issues, plant‐derived extracts could be the best natural alternative medicine with cost‐effectiveness and fewer side effects. Indeed, many recent studies have been reported verifying the anti‐obesity effects of herbal extracts (Kumari et al. 2023; Vrânceanu et al. 2023). In this study, we investigated the effects of sweet flag ( *A. gramineus* ) extract on HFD‐induced obesity and intestinal inflammation.

The genus *Acorus* plant is a medicinal plant that has long been used in India and China (Umamaheshwari and Rekha 2018). Leaves, rhizomes, and essential oils of the genus *Acorus* have been demonstrated to exhibit various beneficial effects, with rhizomes, in particular, being reported to have pharmacological effects such as antibacterial, anti‐inflammatory, antioxidant, and anticancer properties (Rajput, Tonge, and Karuppayil 2014). In the present study, we selected sweet flag ( *A. gramineus* ) and evaluated the in vitro prebiotic activity and in vivo anti‐obesity and intestinal inflammation‐alleviating effects of its extract, SRE.

SRE is mainly composed of sugars and proteins (Table 1). Because of its high sugar content, the active ingredients were predicted to reach the intestine. Therefore, we evaluated the prebiotic activity of SRE using four probiotic strains, including *Lactobacillus* and *Bifidobacterium*, and three probiotic strains isolated from the gut ( *L. gasseri* , *B. longum* , and *B. bifidum* ). The growth and reproduction of probiotics, which strengthen the intestinal barrier and improve immunity, cannot be achieved without prebiotics (You et al. 2022). We compared the prebiotic effects of SRE using inulin, which stimulates beneficial microbiota and improves intestinal integrity and function, as a positive control (Yin et al. 2023). The SRE group exhibited high prebiotic activity scores in a dose‐dependent manner among the four probiotic strains. Furthermore, the SRE 0.5% group showed higher prebiotic activity scores for the four strains than the inulin group. These results indicate that probiotic strains utilize SRE as effectively as they utilize inulin, suggesting that SRE has potential as a prebiotic. We predicted that SRE would affect intestinal integrity and immunity.

In recent years, numerous studies have highlighted intestinal inflammation, damage, and obesity symptoms in relation to HFD (Dang et al. 2023; Di Vincenzo et al. 2024). Therefore, in this study, we evaluated the effects of SRE on symptoms associated with HFD‐induced obesity and intestinal inflammation. We found that SRE administration prevented an increase in body weight and body fat percentage in HFD‐fed mice. The SRE100 group showed decreased HFD‐induced weight gain in the WAT and liver tissues and glucose intolerance. The HFD‐induced obesity model shows symptoms of metabolic dysfunction, such as impaired glucose regulation and an increased serum lipid profile (Nunes‐Souza et al. 2016). In the SRE100 group, the HFD‐induced increase in serum lipids (triglycerides, total cholesterol, and LDL cholesterol) was suppressed. In contrast, the SRE‐administered groups showed increased HDL cholesterol levels in HFD‐fed mice; lower HDL cholesterol levels reflect an increase in the concentration of triglyceride‐rich lipoproteins (Parhofer 2015). Therefore, treatment strategies for obesity should aim to reduce triglyceride‐rich lipoproteins and improve HDL function. In addition, changes in the indirect indicators of fatty liver, such as decreased alanine of ALT and AST, were observed in the SRE100 group. HFD‐induced liver damage is accompanied by an increase in serum ALT and AST levels, as well as an increase in serum lipids (Varani et al. 2022). Serum AST and ALT levels are considered biomarkers and indirect indicators of fatty liver disease (Younossi et al. 2018). SRE administration could also suppress fatty liver symptoms caused by HFD, and further studies are needed in this regard. These findings demonstrated that SRE has beneficial effects on weight gain, glucose intolerance, and serum biomarkers associated with HFD‐induced obesity.

The intestinal barrier is composed of a mucus layer, intestinal epithelial cells, gut microbiota, and tight junctions, and damage to any component of this barrier system increases intestinal permeability, leading to endotoxemia, inflammation, and metabolic disorders (Cani et al. 2009; Rohr et al. 2020). Several studies have shown that HFD can enhance gut permeability and destroy the intestinal barrier system (Chae et al. 2024; Shi et al. 2019). In this study, we also found that HFD significantly induced intestinal permeability and endotoxemia, and verified that SRE administration inhibited these HFD‐induced increases. Additionally, the SRE100 group had a longer colon length and lower colon pH than the HFD group. Colon length is a biomarker associated with intestinal inflammation caused by increased gut permeability (Agranyoni et al. 2024), and a lowered pH in the colon is an indicator that carbohydrate sources have been utilized by the gut microbiota, producing large amounts of short‐chain fatty acids (Yamamura et al. 2023). These results suggest that SRE affects the intestinal barrier by suppressing gut permeability, endotoxemia, and colonic inflammation. Notably, the expression of the tight junction protein ZO‐1 increased after SRE administration in HFD‐fed mice. Tight junction proteins play a key role in maintaining the intestinal barrier and modulating leaky gut symptoms, such as permeability and endotoxemia (Chelakkot, Ghim, and Ryu 2018); these results suggest that SRE administration could alleviate leaky gut syndrome caused by HFD. However, this study has a limitation. Tight junction proteins such as occludin and claudin1 did not significantly increase in the SRE‐administered groups. Therefore, further studies are needed to investigate the relationship between the intestinal environment and SRE.

In this study, we demonstrated the prebiotic effects of SRE on probiotic strains, such as *Lactobacillus* and *Bifidobacterium*. In addition, the administration of 100 mg/kg SRE improved HFD‐induced colonic inflammatory symptoms, such as gut permeability, endotoxemia, colonic shortening, and inhibition of ZO‐1 expression. SRE also attenuated obesity‐related symptoms by inhibiting weight gain, glucose intolerance, and serum lipid biomarkers. Further studies are needed to analyze the functional components of SRE and elucidate their correlation with the treatment of obesity and gut inflammation. Our findings suggest that SRE may help treat obesity and gut inflammation caused by Western‐style diets.

## Author Contributions

**Hye‐Bin Lee:** data curation (lead), formal analysis (lead), methodology (supporting), writing – original draft (lead). **Mi‐Jin Oh:** data curation (supporting), formal analysis (equal). **Hee‐Kyoung Son:** formal analysis (equal). **Miri Park:** formal analysis (equal). **Sang Yoon Choi:** resources (lead). **Jinyoung Hur:** investigation (equal). **Ho‐Young Park:** conceptualization (lead), Supervision (lead), Methodology (lead), Writing – review and editing (lead).

## Conflicts of Interest

The authors declare no conflicts of interest.

## Data Availability

Data will be made available on request.
